# Are Doctors Putting Their Health Last?

**DOI:** 10.7759/cureus.71018

**Published:** 2024-10-07

**Authors:** Vasu Agarwal, Vishnu Prabhakar

**Affiliations:** 1 Respiratory Medicine, Dr. D.Y. Patil Medical College, Hospital and Research Centre, Dr. D.Y. Patil Vidyapeeth (Deemed to be University), Pune, IND

**Keywords:** burnout, doctors, health, obesity, stress

## Abstract

The increasing demands of patient care and the high-stress environment of the medical field significantly impact doctors' physical and mental health. Studies show that physicians face a higher risk of burnout, which leads to emotional exhaustion, reduced sense of accomplishment, and even severe mental health issues such as depression and suicide. This burnout not only affects individual doctors but also significantly compromises patient safety and contributes to a shortage in the physician workforce.

To address these challenges, it is crucial for doctors to prioritize their own health and well-being. This can be achieved through proactive measures such as ensuring adequate rest, reducing administrative burdens, and fostering a supportive environment that encourages open discussion about mental health issues. Hospitals should also implement regular duty rotations, improve doctor-patient ratios, and provide necessary resources to support physician health. Ultimately, promoting a culture where doctors' well-being is valued is essential for a healthier and more effective healthcare system.

## Editorial

As the medical profession grapples with the ever-growing demands of patient care, whether doctors adequately address their health and well-being has become a critical issue.

Doctors, the pillars of the healthcare system are always there to diagnose the patient's mystery illness, stitch you up, or deliver the patient's bundle of joy. Running from patient to patient, dealing with complex cases, and constantly worrying about making the right decision takes a toll. The high-stress nature of the medical field coupled with long working hours can significantly impact both the physical and the mental health of physicians.

Studies show doctors are more likely than others to get sick and burned out as compared to other professions. The consequences of this burnout can impact both the individual and the healthcare system. Physician burnout is associated with a doubled risk of patient safety incidents and contributes to 7% to 10.6% of serious medical errors [1]. Burnout is contributing to a significant shortfall in the physician workforce, with an estimated deficit of 45,000 to 90,000 physicians due to more leaving the field than entering it [1]. On an individual level, it can result in emotional exhaustion, depersonalization, and a diminished sense of personal accomplishment.

As the medical community faces these challenges, doctors must take proactive steps to prioritize their health and well-being. Doctors, like all health professionals, are humans with human needs. Until they propagate role models who exemplify physical and mental well-being rather than complete self-sacrifice the medical profession will continue to be plagued by burnout, depression, and suicide [2]. Harvey et al. highlighted the high prevalence of mental illness and suicide among physicians [3]. An Indian study also identified a high prevalence of overweight and obesity (54%) among doctors [4].

This situation highlights the necessity for change. Hospitals need to give the doctors a fighting chance. Real breaks and not gulping down a sandwich during work hours. Less paperwork and more time with the patients is a doctor's primary aim. Modifiable risk factors like sleep duration and private practice hours warrant prompt attention to improve physician health [4]. Other recommendations for implementation should include regular and consistent duty rotations, improving the standards of communication between the hospital administration and doctors, and ensuring appropriate patient-to-doctor ratio, adequate food, and resting resources for physicians during duty hours. If feeling overwhelmed then both formal and informal support groups should be put in place to openly talk about the mental health challenges without any judgment [5].
